# The coherence time of sunlight in the context of natural and artificial light-harvesting

**DOI:** 10.1038/s41598-022-08693-0

**Published:** 2022-03-31

**Authors:** Berke Vow Ricketti, Erik M. Gauger, Alessandro Fedrizzi

**Affiliations:** Institute of Photonics and Quantum Sciences, School of Engineering and Physical Sciences, Heriot-Watt University, Edinburgh, EH14 4AS UK

**Keywords:** Quantum physics, Biological physics

## Abstract

The suggestion that quantum coherence might enhance biological processes such as photosynthesis is not only of fundamental importance but also leads to hopes of developing bio-inspired ‘green’ quantum technologies that mimic nature. A key question is how the timescale of coherent processes in molecular systems compare to that of the driving light source—the Sun. Across the quantum biology literature on light-harvesting, the coherence time quoted for sunlight spans about two orders of magnitude, ranging from 0.6 to ‘10s’ of femtoseconds. This difference can potentially be significant in deciding whether the induced light-matter coherence is long enough to affect dynamical processes following photoexcitation. Here we revisit the historic calculations of sunlight coherence starting with the black-body spectrum and then proceed to provide values for the more realistic case of atmospherically filtered light. We corroborate these values with interferometric measurements of the complex degree of temporal coherence from which we calculate the coherence time of atmospherically filtered sunlight as $1.12\pm {0.04}\,{\hbox { fs}}$, as well as the coherence time in a chlorophyll analogous filtered case as $4.87\pm {0.21}\,{\hbox { fs}}$.

## Introduction

Photosynthesis, one of the most important biological processes on Earth, has been a primary interest to the quantum biology community ever since the first report of long-lived coherences in the Fenna–Matthews–Olson (FMO) protein trimer in 2007^1^. This subsequently sparked a plethora of theoretical and experimental studies of coherence effects in light-harvesting systems and their potential role in enhancing light-harvesting efficiency^2–10^. Experimentally, two-dimensional electronic spectroscopy (2DES) was utilized to observe long-lived coherences in FMO proteins on $\sim {1}\,{\hbox { ps}}$ timescales^1,5^. However, 2DES uses highly coherent femtosecond lasers, which have low *intr*apulse coherence, but high *inter*pulse coherence within pulse trains. This is fundamentally different from natural sunlight, which is a continuous-wave source with a short coherence time due to its spectral range spanning from the ultraviolet to the mid-infrared, making it strongly incoherent on long-term timescales. These differences have raised questions about to what extent the observed coherences are an artefact of the light source used in the excitation, and if the coherences could occur under natural conditions from incoherent sunlight excitation^11–13^. While the original coherences observed by 2DES are nowadays interpreted as being vibrational rather than electronic^14^, the broader question to what extent the properties of absorbed photons may affect the subsequent evolution of excitonic dynamics remains. For example, recent theoretical work shows that incoherent but polarised illumination can induce excitonic coherences affecting the efficiency of exciton transfer from a donor to an acceptor^15,16^. In any case, both the accurate modelling of natural photosynthetic processes as well as the endeavour to design optimally performing artificial light-harvesting systems^11,17–19^ benefits from a thorough understanding of the coherence properties of natural sunlight.

While work has been done on the spatial coherence properties of sunlight^18,20–22^, there is some disagreement in the community on what value to use for the coherence time of sunlight, with recent literature citing values that stretch two orders of magnitude from 0.6 to “10s” of femtoseconds^13,19,23–36^. Some of the discrepancy originates in a lack of consistent definitions for coherence time, and in a somewhat indiscriminate reliance on theoretic coherence definitions for black-body radiation^37–39^. Indeed, while solar spectroscopy has long been studied^40^, comprehensive experimental studies on the temporal coherence properties of sunlight are surprisingly hard to find in the literature.

In this work, we discuss multiple definitions of coherence time, $\Delta \tau$, to determine what we suggest are the most reasonable. Next, we revisit the literature to survey the range of values quoted for the coherence time of sunlight, $\Delta \tau _{Sun}$, in the quantum biology community and discuss their origins. Then, we validate our recommended $\Delta \tau$ definition with interferometric measurements of sunlight to obtain $\Delta \tau _{Sun}$ and discuss how our experimentally measured values compare to the theoretical calculations previously cited in the literature. Having established values for $\Delta \tau _{Sun}$, we note that sunlight is never unfiltered, but rather progressively filtered as it propagates through Earth’s atmosphere, potentially water in a submerged case, but also more critically, the light harvesting/measurement system itself. Therefore, we investigate the specific case of atmospherically filtered sunlight incident on a plant on Earth’s surface by emulating the absorption spectrum of Chlorophyll-a and Chlorophyll-b, showing that such filtering increases the coherence time in line with analytical modelling of filtered thermal light.

## Theory

Light is an electromagnetic wave which can be represented completely by a complex wavefunction $U(\mathbf{r },t)$^35^. For monochromatic light, the complex wavefunction takes the form of $U(\mathbf{r },t) = U(\mathbf{r })\exp (i\omega t)$, where both the spatial component, $U(\mathbf{r })$, and the temporal component $\exp (i\omega t)$ are deterministic. As such, these components of the wavefunction have perfect periodicity and predictability, and therefore monochromatic light represents a totally coherent light source. By definition, a monochromatic wave has no spectral bandwidth ($\Delta \nu = 0$) and an infinitely long coherence time ($\Delta \tau = \infty$). However, all real world lightwaves have some amount of spectral bandwidth, and thus, some quantifiable $\Delta \tau$, which can span from the order seconds for ultra-stable, single-frequency linewidth lasers to femtoseconds for broadband white light^35,36,39^.

The statistical properties, such as optical intensity and spatial, longitudinal, and temporal coherence of a stationary and ergodic stochastic light source, like natural sunlight, can be determined from the complex wavefunction^20,35^. A comprehensive study of these properties is beyond the scope of this work, but is covered in detail by most standard optics textbooks. In this work, we will be focusing only on temporal coherence, which is represented by the complex degree of temporal coherence (sometimes called the complex autocorrelation function or the complex autocorrelator), $\gamma (t)$. This function acts as a measure of how coherent or incoherent a light source is. For a fixed spatial position $\mathbf{r }$, $\gamma (t)$ can be calculated from the complex wavefunction as

1 $$\begin{aligned} \gamma (t) = \frac{\langle U^*(t') U(t'+t)\rangle }{\langle U^*(t') U(t')\rangle } = \frac{\langle U^*(t') U(t'+t)\rangle }{I_{o}}, \end{aligned}$$

where $*$ is the complex conjugate, $\langle \cdots \rangle$ represents the temporal ensemble average (i.e. $\lim _{T \rightarrow \infty } \frac{1}{2T} \int _{-T}^{T} U^*(t')U(t'+t)dt'$), and $I_{o}$ is the intensity. By definition, $1\ge |\gamma (t)|\ge 0$ and $|\gamma (0)| = 1$. For monochromatic light, $|\gamma (t)| = 1$ for all *t*. Equivalently, it can be shown that $\gamma (t)$ is the Fourier transform of the normalized spectral density of the light source^41^.

The coherence time, $\Delta \tau$, represents the time over which “appreciable amplitude and phase correlations of the light waves persist”^42^. Two of the most common coherence time definitions were introduced by Wolf^38^ and Mandel^37^ as, respectively,

2a $$\begin{aligned} (\Delta \tau _{Wolf})^2&=\frac{\int _{-\infty }^{\infty }t^2|\gamma (t)|^{2}dt}{\int _{-\infty }^{\infty }|\gamma (t)|^{2}dt}, \end{aligned}$$

2b $$\begin{aligned} \Delta \tau _{Mandel}&=\int _{-\infty }^{\infty }|\gamma (t)|^{2}dt. \end{aligned}$$

Both definitions are, effectively, a measure of the width of $|\gamma (t)|$^37^. Specifically, Wolf’s definition is the normalized root-mean-square of the distribution^38^, while Mandel’s can be seen as the “power-equivalent width”^35^. For most simple spectral profiles (e.g. Gaussian or Lorentzian), Eq. (2) yield results that are approximately the same order of magnitude, i.e. $\Delta \tau _{Mandel}/\Delta \tau _{Wolf}\sim 1$. For more complex spectral profiles (e.g. the double-peaked spectral distribution), it has been shown that this relationship does not always hold^42^. Therefore, for anything but a simple spectrum, one must be careful when defining and calculating the coherence time using Eq. (2). Meanwhile, Saleh and Teich^35^ define $\Delta \tau$ as the width of $|\gamma (t)|$, noting that width could have multiple definitions itself, including for example the full-width-at-half-maximum (FWHM) of $|\gamma (t)|$.

**Figure 1:**
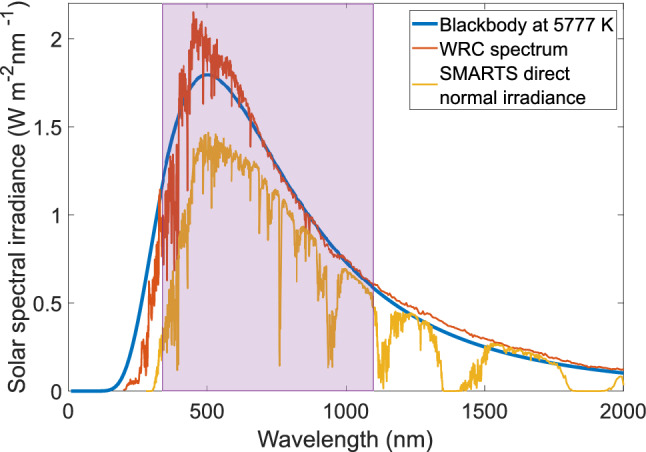
Solar spectral irradiance. The composite solar spectral irradiance curve (orange) compiled by Wehrli^44^ and the Physikalisch-Meteorologisches Observatorium Davos / World Radiation Center (WRC) is often cited as the standard extraterrestrial spectral irradiance. This curve is well approximated by a black-body at 5777  K at 1 AU (blue). The Simple Model of the Atmospheric Radiative Transfer of Sunshine (SMARTS) by Gueymard^45^ and hosted by the National Renewable Energy Laboratory (NREL), is used to model solar spectral irradiance on Earth’s surface. The yellow curve shows the Direct Normal Irradiance in Edinburgh, Scotland in May 2020 using SMARTS version 2.9.5. Compared to the extraterrestrial curve, the SMARTS irradiance shows noticeable atmospheric filtering, including the characteristic absorption bands of $\text {O}_2$, $\text {H}_{2}\text {O}$, and $\text {CO}_2$. The purple window represents the 340–1100 nm spectral responsivity range for the silicon photodetector used to measure $\Delta \tau _{Sun}$ in this work.

With multiple definitions to choose from when calculating $\Delta \tau$, it is not surprising that the community has quoted a wide range of values for $\Delta \tau _{Sun}$. However, when discussing the coherence time of sunlight, the spectral profile of sunlight must be considered, which is complex and cannot be well represented by generic rectangular, Gaussian, or Lorentzian spectral lineshapes. Instead, extraterrestrial sunlight is well approximated by Planck’s Law as a random, continuous-wave black-body source at 5777  K due to its similar spectral irradiance at 1 AU^40,43^, see Fig. 1, and given by $B(\nu )=C\frac{\nu ^3}{\exp (\alpha \nu )-1}$, where $\alpha =h/k_{B}T$, *h* and $k_{B}$ are Planck’s and Boltzmann’s constants, respectively, and *C* is the normalization constant such that $\int _{0}^{\infty }B(\nu )d\nu =1$. The individual optical frequency components of *B*(*v*) may each have a random, time-dependent phase, but this is lost to us due to the numerator of Eq. (1), where the time delayed complex wavefunction is multiplied with its complex conjugate, thereby eliminating any spectral phase information. Mehta^34^ used the Wiener–Khinchin theorem to show that the complex degree of temporal coherence and the normalized spectral density are a Fourier pair, as $\gamma (t) = {\mathscr {F}}^{-1}\{B(\nu )\}=\int _{-\infty }^{\infty }B(\nu )e^{i 2\pi t\nu }d\nu$, and that the coherence time for a black-body at temperature *T* can be calculated using Eq. (2). We can calculate $\Delta \tau _{Sun}$ for a black-body at 5777  K as $\Delta \tau _{Sun, Wolf}={0.58}\,{\hbox { fs}}$ and $\Delta \tau _{Sun,Mandel}={1.28}\,{\hbox { fs}}$. Meanwhile, Hecht^36^, and Saleh and Teich^35^ disregard sunlight’s black-body spectral profile, and instead define the frequency bandwidth of white light in the visible spectrum, $\Delta \nu$, as 0.3e15  Hz and 3.74e14  Hz, respectively. By using $\Delta \tau =1/\Delta \nu$, they then calculate two widely cited $\Delta \tau _{Sun}$ values in the literature: $\Delta \tau _{Sun, Hecht}={3}\,{\hbox { fs}}$ and $\Delta \tau _{Sun, Saleh}={2.67}\,{\hbox { fs}}$.

Clearly, there is neither a single universally accepted expression for calculating $\Delta \tau$, nor an standard value for $\Delta \tau _{Sun}$. Having surveyed the possible definitions of $\Delta \tau$, we proceed to experimentally measuring $\gamma (t)$. Then, once experimental measurements have been completed, we will revisit these definitions to consider which ones best match our empirical findings.

Consider a light beam with intensity $I_{o}$ whose complex wavefunction satisfies our definition from the beginning of this section. The superposition of two copies of this wavefunction result in an interferogram given by $I_{re}(t)=2I_{o}\left[ 1+\text {Re}\{\gamma (t)\}\right]$. This interferogram (whose equation is derived from the interference formula found in standard optics textbooks) is real and time dependent. Since $\gamma (t)$ is complex valued, $I_{re}(t)$ does not capture the information contained in the imaginary part of $\gamma (t)$. Without both the real and imaginary parts of $\gamma (t)$, we are unable to calculate $\Delta \tau$ directly using Eq. (2). Therefore, a recorded interferogram alone contains insufficient information to calculate the coherence time. However, we can recover the complete complex valued $\gamma (t)$ by first recovering the single-beam spectrum.

From Fourier-transform spectroscopy (FTS), we know that we can reproduce the input single-beam spectrum, $B(\nu )$, by taking the inverse Fourier transform of the recorded interferogram as $B(\nu ) = {\mathscr {F}}^{-1}\{I_{re}(t)\}$. Since $I_{re}(t)$ is a real and even function, $B(\nu )$ will also be a real and even function, symmetrically aliased around $\nu = 0$, resulting in negative spectral frequencies such that $B(\nu ) = B(-\nu )$. This spectrum is not realistic and we now assume that the original input spectrum used to record the interferogram, $B_{o}(\nu )$, was real for all positive frequencies and zero otherwise. We can define the unaliased spectrum computed from the interferogram as ${\tilde{B}}(\nu ) = B(\nu )$ for $\nu > 0$. Since ${\tilde{B}}(\nu )$ is a subset of spectral data, it represents the same function as $B_{o}(\nu )$ except with a lower resolution, specifically half the number of data points recorded in $I_{re}(t)$.

${\tilde{B}}(\nu )$ is now a real and odd function whose Fourier transform back to the temporal domain will result in a complex valued function, ${\tilde{\gamma }}(t)={\mathscr {F}}\{{\tilde{B}}(\nu )\}$. ${\tilde{\gamma }}(t)$ represents our complex degree of temporal coherence, except at a reduced resolution with the same number of data points as ${\tilde{B}}(\nu )$. With access to both the real and imaginary parts of ${\tilde{\gamma }}(t)$, we can calculate $\Delta \tau$ using Eq. (2), at the cost of decreased resolution compared to the original recorded interferogram.

## Method

**Figure 2:**
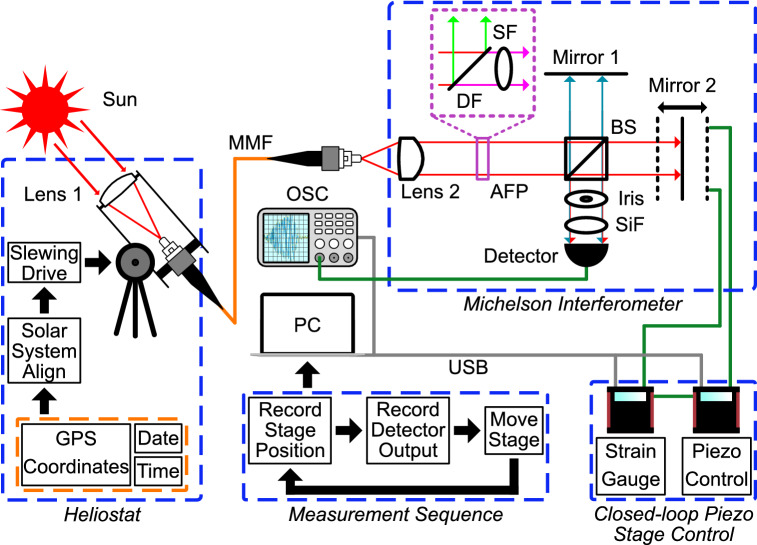
Experimental set-up. Sunlight is captured by a heliostat, consisting of a free-space fibre coupling cage system controlled by a slewing drive with solar system alignment, and coupled into a multimode fibre (MMF). At the MMF output, the sunlight is collimated and is passed into the Michelson Interferometer, where it is split by a non-polarizing 50:50 beamsplitter (BS). Mirror 2 is mounted on a piezo-actuated translation stage with sub-nanometer resolution. The piezo stage is controlled by a strain gauge and piezo controller in closed-loop operation mode. Upon recombination, the light beams are sent through a silicon response-flattening filter (SiF) before being detected by the silicon photodetector. The output of the detector is visualized on an oscilloscope (OSC). Piezo stage translation, stage position readout, and oscilloscope readout are automated by a Matlab measurement sequence running on a PC connected via USB. The analogous filter package (AFP), represented by the purple dashed inset, is removable and was not present for the instrument-filtered data collection. When present, sunlight incident on the dichroic filter (DF) is split based on wavelength: green light is reflected and blocked, magenta light is transmitted and sent to through a 700 nm shortpass filter (SF) before passing to the rest of the Michelson Interferometer.

Figure 2 shows a diagram of the experimental set-up. A heliostat was constructed by mounting a free-space multimode fibre coupler atop a telescope tripod with the ability to track celestial objects. A broadband Michelson interferometer with high temporal resolution was required to observe the interference pattern of sunlight and calculate $\Delta \tau _{Sun}$. The interferometer was constructed to be portable and stable on a small footprint breadboard. A Michelson interferometer is preferable to a spectrometer for measuring coherence time since interferometers offer higher optical throughput (the Jacquinot advantage) and all wavelengths of the light can be measured simultaneously (the Fellgett advantage)^46–48^.

The light beams were split with a beamsplitter cube with a 50:50 splitting ratio for 400–700 nm. Beyond ${700}{\hbox { nm}}$, the splitting ratio performance degrades to $~15\%$ transmission and $~45\%$ reflection at ${1100}{\hbox { nm}}$ due to the influence of the beamsplitter’s AR-coating on light in the infrared portion of the spectrum. The output was detected with an amplified silicon photodetector with switchable gain and a spectral responsivity range of 340–1100 nm. In this state, the input sunlight is subjected to both atmospheric filtering and intrinsic instrument filtering by the experimental set-up. As there are no additional external optical filters applied in this scenario, all references to measurements and results using this set-up will be labeled as “instrument-filtered”.

It is well known in FTS that experimentally recorded interferograms differ from theoretically calculated ones due to phase errors induced by the measurement apparatus. These differences include poorly defined centrebursts and zero path difference (ZPD), as well as asymmetries in the overall interferogram. In FTS, phase errors are corrected by using one of several well established methods; in this work, we implement the Forman phase correction method^48–50^. Once applied to the raw interferograms, these phase corrected interferograms, $I_{PC}(t)$ can then be used to measure $\Delta \tau$.

## Results

**Table 1:** Coherence time of sunlight: experimental vs. theory. The error for the experimental values are calculated as the standard uncertainty of the mean from 30 and 24 measurements collected between August 2019—September 2020 in Edinburgh, Scotland for the instrument-filtered and chlorophyll-filtered cases, respectively. $\Delta \tau _{Wolf}$ and $\Delta \tau _{Mandel}$ are calculated using Eqs. (2a) and (2b), respectively. For the theoretical calculations, a black-body temperature of 5777  K was used. All values have been rounded to two decimal places.

	Instrument-filtered		Chlorophyll-filtered	
	$\Delta \tau _{Wolf}$ (fs)	$\Delta \tau _{Mandel}$ (fs)	$\Delta \tau _{Wolf}$ (fs)	$\Delta \tau _{Mandel}$ (fs)
Experimental $\left( |\gamma (t)|\right)$	$1.12\pm 0.04$	$1.08\pm 0.04$	$4.87\pm 0.21$	$0.92\pm 0.03$
Experimental $\left( \text {Re}\{\gamma (t)\}\right)$	$1.13\pm 0.03$	$0.61\pm 0.02$	$4.49\pm 0.15$	$0.61\pm 0.02$
Theory (Sigmoid Model)	1.13	1.89	5.00	3.27

**Figure 3:**
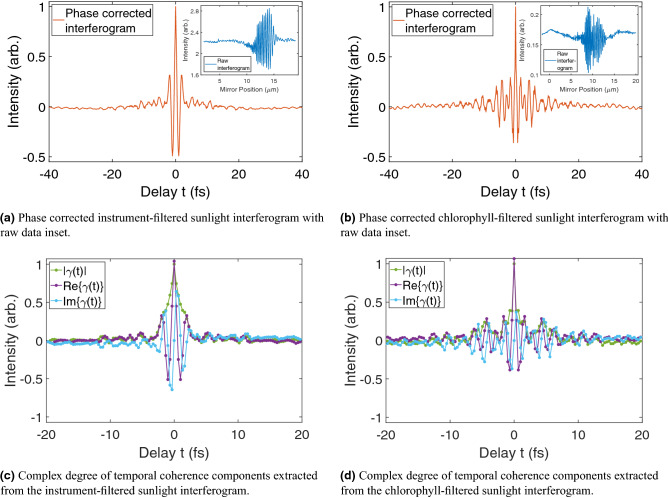
Instrument and chlorophyll-filtered sunlight interferograms. The raw instrument-filtered interferogram was recorded on August 23rd, 2019, at 11:36 and the raw chlorophyll-filtered interferogram was recorded on September 18th, 2020, at 12:54. All interferograms were recorded in Edinburgh, Scotland between August 2019 and September 2020. (**a**,**b**) both show well defined centre bursts and strong symmetry around the ZPD due to successful phase correction using the Forman method. The interferogram in (**b**) shows more prominent sidelobe oscillations compared to (**a**) due to the additional filtering by the chlorophyll analogous filter package. Raw data for each phase corrected interferogram can be seen in the respective inset. The complex degree of temporal coherence components of (**c**,**d**) have lower resolution compared to the phase corrected interferograms of (**a**,**b**), respectively, due to the method by which the $\gamma (t)$ is extracted from the single-beam spectrum.

For each phase corrected sunlight interferogram, $\Delta \tau _{Wolf}$ and $\Delta \tau _{Mandel}$ were calculated using two different methods. In the first method, $I_{PC}(t)$ was Fourier transformed back into the single-beam spectrum to extract $\gamma (t)$. The $\Delta \tau _{Wolf}$ and $\Delta \tau _{Mandel}$ results calculated using this method are labelled as “$|\gamma (t)|$” in Table 1. In the second method, coherence times were calculated directly from $I_{PC}(t)$ without extracting the complex-valued $\gamma (t)$. This is equivalent to replacing $|\gamma (t)|$ with $\text {Re}\{\gamma (t)\}$ in Eq. (2), and allows for a direct comparison to understand the influence of $I_{im}$ on the $\Delta \tau _{Wolf}$ and $\Delta \tau _{Mandel}$ calculations. In this case, these values are labelled as “$\text {Re}\{\gamma (t)\}$” in Table 1. Table 1 provides the mean coherence time value in femtoseconds from 30 experimental measurements for the instrument-filtered case. Figure 3 shows an example of phase corrected sunlight interferograms and the extracted $\gamma (t)$ components.

Theoretical calculations for $\Delta \tau _{Wolf}$ and $\Delta \tau _{Mandel}$ assume a truncated black-body at 5777  K. In all cases, wavelength cut-offs are modelled as sigmoidal filter functions of the form $\varsigma (\lambda ) = \frac{1}{1+e^{-c_{1}(\lambda -c_{2})}}$. Here $c_{1}$ is the slope parameter which dictates the steepness of the wavelength cut-off and $c_{2}$ is the centring parameter which is the wavelength at which the filter function yields 50% transmission. Sigmoid functions were chosen as they do not have sharp cut-offs or discontinuities, since the hard cut-offs of a step filter function are neither practically realistic nor computationally convenient as they lead to undesirable artefacts upon Fourier transforming and applying to Eqs. (2a) and (2b). Sigmoidal filter functions were stacked to emulate bandpass filters, and the filtered spectrum is given by multiplying the input spectrum (e.g. a blackbody at ${5777}\,{\hbox { K}}$) by the filter function. The instrument-filtered results assume $c_{2}$ parameters centred at ${340}{\hbox { nm}}$ and ${1100}{\hbox { nm}}$, the wavelength range of our photodetector, and $c_{1}$ parameters such that the cut-off is sufficiently steep.

While discussing the theoretical or actual coherence time of sunlight at Earth’s surface is interesting, this cannot be directly applied to plants without first regarding the additional filtering imposed by photosynthetic structures. Land plants and green algae on Earth capture sunlight using their pigment-protein complexes (PPCs), which are comprised of a number of Chlorophyll-a and Chlorophyll-b molecules^30,51,52^. Each molecule possesses a specific, well defined, and well known absorption spectrum^53^. Specifically, both Chlorophyll-a and Chlorophyll-b have twin peak absorption spectra in the visible wavelength range of 400–700 nm. When taken together, the combined spectrum absorbs from approximately 400–500 nm and 600–700 nm, with negligible absorption below 400 nm (the ultraviolet region), between 500–600 nm, and above 700 nm (the near-infrared region).

**Figure 4:**
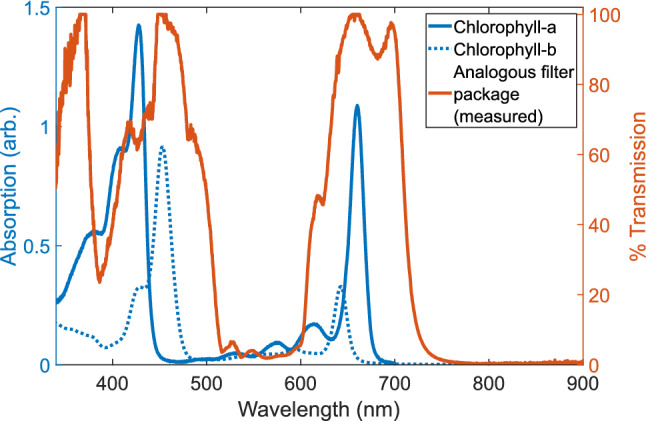
Chlorophyll Filtering. The absorption profile of Chlorophyll-a and Chlorophyll-b in diethyl ether^54,55^ is emulated by the combination of a $45^{\circ }$ magenta dichroic filter and a 700 nm shortpass filter. The filter package effectively cuts out the green light band from 500–600 nm and the near-IR above 700 nm. It is important to note that the dip in measured transmission percentage between 370–450 nm is not due to filtering by the filter package, but rather a limitation of the light sources used to measure the transmission. It is reasonable to expect the actual transmission percentage to be near 100%, based on the transmission specifications of the components used.

To emulate the combined Chlorophyll-a and Chlorophyll-b absorption profile, additional filters were added to the Michelson interferometer. The first was a magenta dichroic filter with a transmission window of 500–600 nm (green) and reflection windows of 400–500 nm and 600–700 nm (magenta) when positioned at $45^{\circ }$ with respect to the incident light. The second filter was a 700 nm shortpass filter to cut off wavelengths in the near-infrared. Combined, these two filters effectively emulate the absorption bands of Chlorophyll-a and Chlorophyll-b in diethyl ether^54,55^, as shown in Fig. 4. Sunlight interferograms recorded with this filter package will be designated as “chlorophyll-filtered” from here on. Table 1 provides the mean coherence time value from 24 experimental measurements for the chlorophyll-filtered case.

Theoretical models of the chlorophyll-filtered case were also developed using sigmoidal filter functions, with $c_{2}$ cut-off parameters at ${340}{\hbox { nm}}$, ${500}{\hbox { nm}}$, ${625}{\hbox { nm}}$, and ${710}{\hbox { nm}}$. In this case, ${500}{\hbox { nm}}$, ${625}{\hbox { nm}}$, and ${710}{\hbox { nm}}$ were chosen as they correspond to the $\sim 50\%$ transmission levels in the analogous filter package (see Fig. 4), and $c_{1}$ parameters were chosen to emulate the slopes at each of these cut-offs.

## Discussion

In the instrument-filtered case, the measurement of $\Delta \tau _{Wolf}=1.12\pm {0.04}\,{\hbox { fs}}$ using $|\gamma (t)|$ agrees with the theoretical sigmoid modelled calculations of 1.13  fs. Similarly for the chlorophyll-filtered case, $\Delta \tau _{Wolf}=4.87\pm {0.21}\,{\hbox { fs}}$ using $|\gamma (t)|$ agrees with the theoretical calculation of 5.00  fs. In both experimental and theoretical results, we see the $\Delta \tau _{Wolf}$ coherence time increasing when moving from the instrument-filtered case to the chlorophyll-filtered case, an expected result due to the increased filtering. These $\Delta \tau _{Wolf}$ results fall within the 0.6 to ‘10s’ of femtoseconds range often cited in literature for $\Delta \tau _{Sun}$ and suggest that Wolf’s Eq. 2a is suitable for use with our sunlight interferograms.

Conversely, calculations of $\Delta \tau _{Mandel}$ using Eq. 2b did not yield expected results, with all experimental $\Delta \tau _{Mandel}$ measurements failing to agree with their respective theoretical calculations. For both the instrument and chlorophyll-filtered cases, the theoretical values of $\Delta \tau _{Mandel}={1.89}\,{\hbox { fs}}$ and $\Delta \tau _{Mandel}={3.27}\,{\hbox { fs}}$, respectively, fall far outside the error bounds of the experimental calculations for $\Delta \tau _{Mandel}$. Furthermore, the experimental results would suggest that the additional filtering of the chlorophyll-filtered case would *decrease* the coherence time, rather than increase it. Therefore, Mandel’s Eq. 2b is not suitable for use with our sunlight interferograms.

Comparing the $\Delta \tau _{Wolf}$ calculations which used $|\gamma (t)|$ against those that used $\text {Re}\{\gamma (t)\}$, we see that there is little difference in the instrument-filtered case, but a larger difference in the chlorophyll-filtered case. This difference is most likely due to the more complex shape of $|\gamma (t)|$ in the chlorophyll-filtered case, and as such, $\text {Im}\{\gamma (t)\}$ has a larger contribution than in the instrument-filtered case. Regardless, we see closer agreement between the experimental and theoretical results when calculating $\Delta \tau _{Wolf}$ with $|\gamma (t)|$. This agreement suggests that $I_{PC}(t)$ alone, without any transformation into the single-beam spectrum, is insufficient for accurately calculating the coherence time of the light, as expected. As previously mentioned, the method of extracting $|\gamma (t)|$ from the single-beam spectrum required halving the number of data points, reducing the resolution of $|\gamma (t)|$. The resolution could be improved by sampling the raw interferograms at larger delays, but this exceeded the available range of our piezo stage.

The absorption bands of Chlorophyll depend on the electrostatic interactions, with the Qx and Qy transitions in Chlorophyll-a being the two lowest absorption bands with orthogonal transition dipole moments^56,57^. The absorption spectra of these transitions falls in the 600–700 nm, and by applying our sigmoidal filter method with these cut-offs, we can calculate the filtered coherence time of a blackbody at 5777 K using Wolf’s and Mandel’s definitions as: $\Delta \tau _{Wolf,QxQy} = {6.34}\,{\hbox { fs}}$ and $\Delta \tau _{Mandel,QxQy} = {12.58}\,{\hbox { fs}}$, respectively. If we expand our scope to include both Chlorophyll- and Chlorophyll-b, we can predict the coherence time of chlorophyll-filtered light by producing a toy model of the filter function of a combined Chlorophyll-a/b system ($Chl_{AB}(\lambda )$). Consider the absorption spectra of Chlorophyll-a ($Chl_{A}(\lambda )$) and Chlorophyll-b ($Chl_{B}(\lambda )$) (blue curves in Fig. 4). If we were to assume the total absorption spectrum of a combined system is the linear combination of the two sub-systems, such that $Chl_{AB}(\lambda ) = Chl_{AB}(\lambda )+ Chl_{B}(\lambda )$, and then scaled $Chl_{AB}(\lambda )$ such that the maximum value of is 100, we can essentially treat this combined spectrum as a Chlorophyll-a/b filter function of transmission percentage with respect to wavelength (similar to the orange curve of Fig. 4). Furthermore, we will assume $Chl_{AB}(\lambda ) = 0$ for $\lambda < {340}{\hbox { nm}}$ and $\lambda > {700}{\hbox { nm}}$. If we then apply this filter function to a black-body at 5777  K, we can calculate the coherence time for the toy model filter function of a Chlorophyll-a/b system as $\Delta \tau _{Wolf,ChlAB}={11.37}\,{\hbox { fs}}$ and $\Delta \tau _{Mandel,ChlAB}={4.94}\,{\hbox { fs}}$.

While many photosynthetic organisms utilize Chlorophyll-a and Chlorophyll-b in their PPCs, other organisms, such as purple bacteria, green sulphur bacteria, and brown and red algae, do not. These organisms use other molecules for photosynthesis, including Chlorophyll-c, Bacteriochlorophyll-a and Bacteriochlorophyll-b^51^. Since these molecules each have individual absorption spectra which differ from those of Chlorophyll-a and Chlorophyll-b, the $\Delta \tau _{Sun}$ values discussed in this paper only relate to plants and green algae. Different filter packages could be utilized to try and match the absorption spectra of other organisms or Chlorophyll/Bacteriochlorophyll combinations, and therefore, measure the $\Delta \tau _{Sun}$ which would be observed by them. Indeed, a study of the observed $\Delta \tau _{Sun}$ values for different types of organisms, based on their own PPC absorption spectral profile, would be novel application of this work, and would be of particular interest to the quantum biology community if surprisingly large coherence times were found in specific systems. Furthermore, this work focused on filtered sunlight on Earth’s surface, but does not consider photosynthesis underwater. It is well known that many photosynthetic organisms, including green sulphur bacteria, have been found in low light environments, and as deep 145  m underwater^58^. A study of the coherence time of sunlight in underwater environments could also be of interest.

Finally, it is straightforward to model networks of coupled chromophores interacting with a radiation field in the fully coherent limit (using a coherent driving term in the Hamiltonian) and in the fully incoherent limit (i.e. coupling to a thermal radiation bath via the standard quantum optical master equation)^30^. By contrast, the partially coherent case (e.g. incoherent sunlight progressively beginning filtered by Earth’s atmosphere and Chlorophyll molecules) poses a bigger challenge. Work has been done to show how to deal with weak excitation (e.g. sunlight), linking the ensuing dynamics to the autocorrelation function of a semi-coherent light field^59,60^. The results of our work could inform the parameters going into this, or related modelling approaches.

## Conclusion

Proper characterisation of the coherence properties of sunlight is essential when modelling photosynthetic complexes or developing artificial light-harvesting systems. Inconsistencies in quoted values for the coherence time of sunlight abound and span over an order of magnitude. In this work, we examined the origins of these various values and how they were calculated. Then, we established a baseline method for calculating $\Delta \tau$ directly from an experimentally measured interferogram and performed interferometric experiments to measure $\gamma (t)$. Using the complex degree of temporal coherence, we calculate $\Delta \tau _{Sun}$ using Wolf and Mandel’s equations. We find that Wolf’s coherence time definition as the normalized root-mean-square width of the complex degree of temporal coherence yields the most reliable results, and we calculate coherence times of $1.12\pm {0.04}\,{\hbox { fs}}$ in the instrument-filtered case and $4.87\pm {0.21}\,{\hbox { fs}}$ in the chlorophyll-filtered case. By comparison, Wolf’s definition using theoretical models of a black-body at ${5777}\,{\hbox { K}}$ yield coherence times of ${0.58}\,{\hbox { fs}}$ in the unfiltered case, ${1.13}\,{\hbox { fs}}$ in the instrument-filtered case, and ${5.00}\,{\hbox { fs}}$ in the chlorophyll-filtered case. We show that Mandel’s definition for coherence time cannot reliably be applied to more complex spectra of the type relevant for photosynthesis, and we discuss possible future calculations with different spectral filtering cases which may be useful for the community. It is our hope that this work can act as a useful reference in the future.
